# Optimizing prevention of HIV mother to child transmission: Duration of antiretroviral therapy and viral suppression at delivery among pregnant Malawian women

**DOI:** 10.1371/journal.pone.0195033

**Published:** 2018-04-03

**Authors:** Maganizo B. Chagomerana, William C. Miller, Jennifer H. Tang, Irving F. Hoffman, Bryan C. Mthiko, Jacob Phulusa, Mathias John, Allan Jumbe, Mina C. Hosseinipour

**Affiliations:** 1 UNC Project-Malawi, Kamuzu Central Hospital, Lilongwe, Malawi; 2 Division of Epidemiology, College of Public Health, The Ohio State University, Columbus, OH, United States of America; 3 Department of Obstetrics and Gynecology, The University of North Carolina at Chapel Hill, Chapel Hill, NC, United States of America; 4 Department of Medicine, The University of North Carolina at Chapel Hill, Chapel Hill, NC, United States of America; Boston University, UNITED STATES

## Abstract

**Background:**

Effective antiretroviral therapy during pregnancy minimizes the risk of vertical HIV transmission. Some women present late in their pregnancy for first antenatal visit; whether these women achieve viral suppression by delivery and how suppression varies with time on ART is unclear.

**Methods:**

We conducted a prospective cohort study of HIV-infected pregnant women initiating antiretroviral therapy for the first time at Bwaila Hospital in Lilongwe, Malawi from June 2015 to November 2016. Multivariable Poisson models with robust variance estimators were used to estimate risk ratios (RR) and 95% confidence intervals (CI) of the association between duration of ART and both viral load (VL) ≥1000 copies/ml and VL ≥40 copies/ml at delivery.

**Results:**

Of the 252 women who had viral load testing at delivery, 40 (16%) and 78 (31%) had VL ≥1000 copies/ml and VL ≥40 copies/ml, respectively. The proportion of women with poor adherence to ART was higher among women who were on ART for ≤12 weeks (9/50 = 18.0%) than among those who were on ART for 13–35 weeks (18/194 = 9.3%). Compared to women who were on ART for ≤12 weeks, women who were on ART for 13–20 weeks (RR = 0.52; 95% CI: 0.36–0.74) or 21–35 weeks (RR = 0.26; 95% CI: 0.14–0.48) had a lower risk of VL ≥40 copies/ml at delivery. Similar comparisons for VL ≥1000 copies/ml at delivery showed decrease in risk although not significant for those on ART 13–20 weeks.

**Conclusion:**

Longer duration of ART during pregnancy was associated with suppressed viral load at delivery. Early ANC attendance in pregnancy to facilitate prompt ART initiation for HIV-positive women is essential in the effort to eliminate HIV vertical transmission.

## Introduction

Among pregnant women, elevated viral loads in the plasma or genitourinary tract increase the risk of HIV mother-to-child-transmission (MTCT).[1–3] Without any intervention, high viral loads have been associated with a 20–25% risk of vertical HIV transmission.[4] Effective antiretroviral therapy (ART) reduces viral load in the plasma[5, 6] and cervicovaginal fluid[6] by blocking HIV replication in people living with HIV infection, including HIV-infected pregnant women. As a result, ART has been very effective in reducing both vertical [7, 8] and horizontal HIV transmission to a ≤2% vertical transmission risk among women with a plasma viral load (VL) <1000 copies/ml.[9–11]

Persistently high levels of VL in HIV-infected persons on ART is often an indication of poor adherence and/or treatment failure that can subsequently lead to the development of an HIV drug resistant (HIVDR) strain.[12] For HIV-infected pregnant women, the HIVDR strain can be transmitted to the infant, resulting in impaired efficacy of ART in both mother and child.[13] Failure to achieve adequate viral suppression during pregnancy can also lead to challenges in ART treatment for the infant.

In Malawi, Option B+ has dramatically increased uptake of the PMTCT program.[14] In Option B+ programs, HIV-infected pregnant women who are not on ART are started on ART during pregnancy and continued for life regardless of CD4 cell count or WHO clinical stage.[15, 16] As some women present late in pregnancy for their first antenatal care (ANC) visit and ART initiation, these women may not achieve viral suppression by the time of delivery if duration of ART and ART adherence is insufficient.

Therefore, our study’s primary objective was to analyze data from newly-diagnosed HIV-infected pregnant women who started ART at a large urban hospital in Lilongwe, Malawi and evaluate the association between duration on ART and viral suppression by the time of delivery. Our secondary objective was to evaluate the effect of ART adherence on this association.

## Methods

### Study design, population and setting

We conducted a prospective cohort study of HIV-infected pregnant women initiating ART for the first time under the Option B+ program at Bwaila Hospital in Lilongwe, Malawi, from June 2015 to November 2016. Participants enrolled in this study were recruited from ANC clinic and included women who were presenting for their first ANC visit for the current pregnancy.

As part of routine care, all pregnant women attending their first ANC for the current pregnancy were interviewed to determine their HIV status and ART exposure. Information collected during the interview was cross-checked with documentation in the participant’s personal health passport, a government-issued document that contains information on general history, diagnoses, treatments, antenatal consultations, and deliveries. Women with unknown or negative HIV status were tested for HIV using two rapid tests to establish or confirm their HIV status. All HIV-infected pregnant identified were enrolled in the national PMTCT program. HIV-infected women who were already on ART were continued on their therapy while those who were not on ART or were exposed only to single-dose nevirapine (NVP) or zidovudine (ZDV) during a previous pregnancy were initiated on ART immediately.

All confirmed HIV-infected women aged ≥16 years who were ART-naïve or exposed only to single-dose nevirapine (NVP) or zidovudine (ZDV) during a previous pregnancy were eligible for the study. After providing written informed consent, all eligible women were enrolled and started on first-line ART regimen with tenofovir, lamivudine and efavirenz (TDF/3TC/EFV) at the first ANC visit.

This study was approved by the National Health Sciences Research Committee of Malawi and the Institutional Review Board at the University of North Carolina at Chapel Hill.

### Follow-up and data collection

We followed all enrolled women from the first day of ART initiation (or antenatal care) until delivery. The follow-up schedule followed standard ANC guidelines in Malawi; all enrolled women were seen monthly after the initial ANC visit until delivery. In addition to clinical information, we also collected information on ART adherence based on reported number of missed pills, number of pills brought to the clinic, number of pills left at home, and number of pills dispensed during the scheduled visits.

### Laboratory investigations

CD4 cell count testing was conducted at the clinic site during the initial ANC visit using Becton Dickinson FACScounts. HIV RNA was measured at baseline, 6 months (for those whose initial ANC visit was in the first trimester), and at labor and delivery. HIV RNA testing was conducted at the UNC Project-Malawi clinical research laboratory using the Abbott M2000 system. Women who had detectable viral load were offered ART adherence counselling.

### Variable definitions and classification

#### Main exposure

The main exposure was duration of ART during the current pregnancy measured in weeks and derived from the time elapsed between ART start date (first ANC visit date) and delivery date. Women were assigned to one of three categories for ART duration: 1) ≤12 weeks, 2) 13–20 weeks, or 3) 21–35 weeks.

#### Main outcome

The main outcome was HIV VL level at delivery based on VL tests done within 14 days of delivery date (within 14 days before or after delivery). We looked at viral suppression at two cut-off points, 1000 copies/ml and 40 copies/ml (40 copies/ml is the level of detection at the UNC Project-Malawi laboratory). We classified a woman as having failed to achieve sufficient viral suppression and complete viral suppression at delivery if VL was ≥ 1000 copies/ml and ≥ 40 copies/ml, respectively.

#### Confounding and effect measure modification

Prior to analysis, we identified age, body mass index, CD4 cell count, VL, education, marital status, parity, and HIV WHO clinical stage at baseline as potential confounders of the relationship between duration of ART and viral suppression at delivery based on clinical relevance and literature review. All confounders except education (no school or primary, secondary or tertiary), marital status (single, married, or divorced/widowed), WHO clinical stage (Stage1 or Stages 2 & 3) were modeled as continuous or ordinal to minimize sparse data. All predetermined potential confounders were included in all multivariable models.

Adherence to ART affects viral suppression regardless of the period that an individual has been on therapy. We identified ART adherence as a potential effect measure modification (EMM) factor of the relationship between duration of ART and viral suppression at delivery. To ascertain if an individual was adherent to ART, we collected the self-reported number of pills missed yesterday, 2 days ago, 2 weeks ago, and 30 days ago. In addition, we recorded the number of pills brought to the clinic, self-reported number of pills left at home, and number of pills dispensed during every clinic visit. The number of pills brought to the clinic and self-reported number of pills left at home was used to verify the self-reported number of pills missed. We defined ART non-adherence as a ratio of the number of pills missed to the number of pills dispensed during the pregnancy; a ratio of 0 represents full adherence, and a ratio of 1 represents complete non-adherence. We dichotomized ART non-adherence as: 1) good adherence (ART non-adherence ratio = 0–5%), and 2) poor adherence (ART non-adherence ratio >5%).

### Statistical analyses

We used univariable logistic regression models to assess if there was an association between baseline characteristics and both VL ≥1000 copies/ml and VL ≥40 at delivery. Pearson correlation coefficients were used to assess for correlations between BMI, age, CD4 cell count, VL, and WHO clinical stage. In the primary analysis, we used multivariable Poisson models with robust variance estimators to estimate risk ratios (RR) and 95% confidence intervals (CI) of the association between categories of duration of ART and both VL ≥1000 copies/ml and VL ≥40 copies/ml at delivery. In the analysis exploring EMM for ART adherence, we collapsed duration of ART into two categories: 1) ≤12 weeks, and 2) 13–35 weeks to minimize sparse data. In a secondary analysis, we used multivariable Poisson models with robust variance estimators to estimate RRs and 95% CIs for the association between duration of ART as a continuous measure (in weeks) and both VL ≥1000 copies/ml and VL ≥40 copies/ml at delivery.

All analyses were performed using Stata software, version 14.1 (StataCorp LP, College Station, Texas, USA).

## Results

A total of 299 women were enrolled during the study period. At first ANC visit, 266 (89%) were married, and 139 (47%) had already given birth at least twice (Table 1). The median age of the women was 26.7 years (Interquartile rage (IQR): 22.9–30.2), and median gestational age was 22.1 weeks (IQR: 18.1–26.3). The median duration on ART prior to delivery was 17 weeks (IQR: 13–21). Women who were on ART for >12 weeks were more likely to have secondary or tertiary education (OR = 1.86; 95% CI: 0.99, 3.49 for 13–20 weeks and OR = 2.55; 95% CI: 1.27–5.09 for 21–35 weeks) when compared to women who were on ART for ≤12 weeks. Similarly, women who were on ART for >12 weeks were more likely to be nulliparous or primiparous (OR = 1.46; 95%CI: 0.80–2.66 for 13–20 weeks and OR = 2.35; 95% CI: 1.20–4.61 for 21–35 weeks) than women who were on ART for ≤12 weeks. Otherwise, at enrollment the women in the three ART duration categories were similar in age, BMI, marital status, CD4 cell count, and VL.

**Table 1:** Characteristics of the participants at enrollment.

Characteristic		Total N = 299	Duration of ART at Delivery		
			≤ 12 weeks N = 68	13–20 weeks N = 129	21–35 weeks N = 75
Age (years)					
	Median (IQR)	26.7 (22.9, 30.2)	27.3 (22.2, 31.0)	26.9 (23.0, 30.9)	26.2 (22.1, 29.5)
BMI (kg/m^2^)					
	Median (IQR)	23.8 (21.9, 26.6)	23.9 (22.2, 26.7)	23.9 (21.5, 26.7)	23.5 (22.0, 25.6)
Gestational age (weeks)					
	Median (IQR)	22.1 (18.1, 26.3)	27.9 (22.8, 31.4)	23.7 (21.3, 26.0)	17.0 (13.9, 19.3)
Education [n (%)]					
	No school or primary	172 (57.7)	47 (70.2)	72 (55.8)	36 (48.0)
	Secondary or tertiary	126 (42.3)	20 (29.8)	57 (44.2)	39 (52.0)
Marital Status [n (%)]					
	Single	10 (3.3)	4 (5.9)	3 (2.3)	1 (1.3)
	Married	266 (89.0)	58 (85.3)	118 (91.5)	68 (90.7)
	Divorced/widowed	23 (7.7)	6 (8.8)	8 (6.2)	6 (8.0)
Parity [n (%)]					
	Nulliparity	53 (17.9)	13 (19.4)	16 (12.6)	18 (24.0)
	Primiparity	104 (35.1)	14 (20.9)	47 (37.0)	28 (37.3)
	Multiparity	139 (47.0)	40 (59.7)	64 (50.4)	29 (38.7)
CD4 count (cells/mm^3^)					
	Median (IQR)	350 (231, 517)	375 (236, 530)	326 (232, 537)	354 (227, 502)
Viral load (copies/ml)					
	Median (IQR)	15627 (3258, 48,237)	16033 (4250, 42614)	14692 (2642, 52426)	15902 (3744, 41763)
WHO clinical stage [n (%)]					
	Stage 1	284 (95.0)	62 (91.2)	122 (94.6)	73 (97.3)
	Stage 2 or 3	15 (5.0)	6 (8.8)	7 (5.4)	2 (2.7)

Note: 8 Missing BMI, 1 missing education, 2 missing parity, 5 missing baseline CD4 count, 13 missing baseline viral load, and 27 missing delivery information (includes LTFU).

Of the 299 women enrolled, 252 women (84.3%) had a VL performed at the time of delivery. We did not have information for viral load at delivery for 47 women who were either lost to follow before delivery (9 women) or delivered at home /at another hospital and did not return for viral load testing within 14 days of delivery (38 women). Among the 252 women who had VL test results at delivery, 40 (15.9%) and 78 (31%) had VL ≥1000 copies/ml and VL ≥40 copies/ml, respectively (Table 2). Women who had VL ≥40 copies/ml at delivery had a lower CD4 count (odds ratio (OR) = 0.82; 95% CI: 0.71–0.94)) and higher VL (OR = 1.96; 95% CI: = 1.36–2.83) at enrollment than those who had VL <40 copies/ml at delivery. Women who had VL ≥1000 copies/ml at delivery had lower CD4 count (OR = 0.82; 95%CI: 0.68–0.98) but similar VL (OR = 1.32; 95% CI: 0.87–2.00) at enrollment to those who had VL <1000 copies/ml at delivery.

**Table 2:** Predictors of viral suppression at the time of delivery.

Characteristic		Viral load ≥1000 copies/ml			Viral load ≥40 copies/ml		
		Yes (N = 40)	No (N = 212)	OR (95% CI)	Yes (N = 78)	No (N = 174)	OR (95% CI)
Age							
	Median (IQR)	25.1 (22.3, 29.7)	27.0 (22.9, 31.0)	0.96 (0.90, 1.03)	25.8 (22.3, 29.5)	27.2 (23.0, 31.3)	0.96 (0.91, 1.02)
BMI							
	Median (IQR)	24.9 (22.3, 28.0)	23.9 (21.8, 26.6)	1.06 (0.97, 1.15)	24.3 (22.2, 26.7)	23.6 (21.6, 26.6)	1.05 (0.97, 1.12)
Education [n (%)]							
	No school or Primary	24 (61.5)	120 (56.6)	1.00	49 (63.6)	95 (54.6)	1.00
	Secondary or Tertiary	15 (38.5)	92 (43.4)	0.82 (0.40, 1.64)	28 (36.4)	79 (45.4)	0.69 (0.40, 1.19)
Marital Status [n (%)]							
	Single	2 (5.0)	6 (2.8)	1.82 (0.35, 9.38)	4 (5.1)	4 (2.3)	2.37 (0.58, 9.77)
	Married	35 (87.5)	191 (90.1)	1.00	67 (85.9)	159 (91.4)	1.00
	Divorced or Widowed	3 (7.5)	15 (7.1)	1.09 (0.30, 3.97)	7 (9.0)	11 (6.3)	1.51 (0.56, 4.06)
Parity [n (%)]							
	Nulliparity	6 (15.0)	37 (17.6)	1.00	16 (20.5)	27 (15.7)	1.00
	Primiparity	14 (35.0)	68 (32.4)	1.27 (0.45, 3.58)	20 (25.6)	62 (36.0)	0.54 (0.25, 1.21)
	Multiparity	20 (50.0)	105 (50.0)	1.17 (0.44, 3.15)	42 (53.9)	83 (48.3)	0.85 (0.42, 1.76)
CD4 cell count (/100)							
	Median (IQR)	3.0 (1.7, 4.9)	3.6 (2.6, 5.2)	0.82 (0.68, 0.98)	3.0 (2.0, 4.8)	3.8 (2.6, 5.3)	0.82 (0.71, 0.94)
Viral load (log_10_)							
	Median (IQR)	4.4 (3.8, 4.7)	4.2 (3.5, 4.7)	1.32 (0.87, 2.00)	4.5 (3.9, 4.6)	4.1 (3.4, 4.6)	1.96 (1.36, 2.83)
WHO clinical stage [n (%)]							
	Stage 1	36 (90.0)	202 (95.3)	1.00	73 (93.6)	165 (94.8)	1.00
	Stage 2 or 3	4 (10.0)	10 (4.7)	2.24 (0.67, 7.54)	5 (6.4)	9 (5.2)	1.26 (0.41, 3.88)

Note: 5 Missing BMI, 1 missing education, 2 missing parity, 2 missing baseline CD4 count, and 11 missing baseline viral load

More women on ART for ≤12 weeks had VL ≥1000 copies/ml at delivery (15/56 = 26.8%), compared to women on ART for 13–20 weeks (19/125 = 15.2%) or 21–35 weeks (6/71 = 8.5%). Over half (32/56 = 57.1%) of the women on ART for ≤12 weeks had VL ≥40 copies/ml at delivery, compared to 28.0% (35/125) for 13–20 weeks and 15.5% (11/71) for 21–35 weeks. Of the 56 women on ART for ≤12 weeks, 12 were on ART for 0–6 weeks and 44 were on ART for 7–12 weeks (Table 3). Among the 12 women who were on ART 0–6 weeks, 2 (16.6%) had VL ≥1000 copies/ml and 5 (41.7%) had VL ≥40 copies/ml at delivery. Women who were on ART 7–12 weeks showed a similar trend in VL at delivery with 13 (29.5%) having VL ≥1000 copies/ml and 27 (61.4%) having VL ≥40 copies/ml.

**Table 3:** Distribution of women who started ART ≤ 12 weeks.

	Viral load ≥1000 copies/ml		Viral load ≥40 copies/ml	
Duration of ART	Yes N (%)	No N (%)	Yes N (%)	No N (%)
0–3 weeks	0 (0.0)	1 (100)	1 (100)	0 (0.0)
4–6 weeks	2 (18.2)	9 (81.8)	4 (36.4)	7 (63.6)
7–9 weeks 10–12 weeks	3 (27.3)	8 (72.7)	9 (81.8)	2 (18.8)
	10 (30.3)	23 (69.7)	18 (54.5)	15 (45.5)

**Fig 1:**
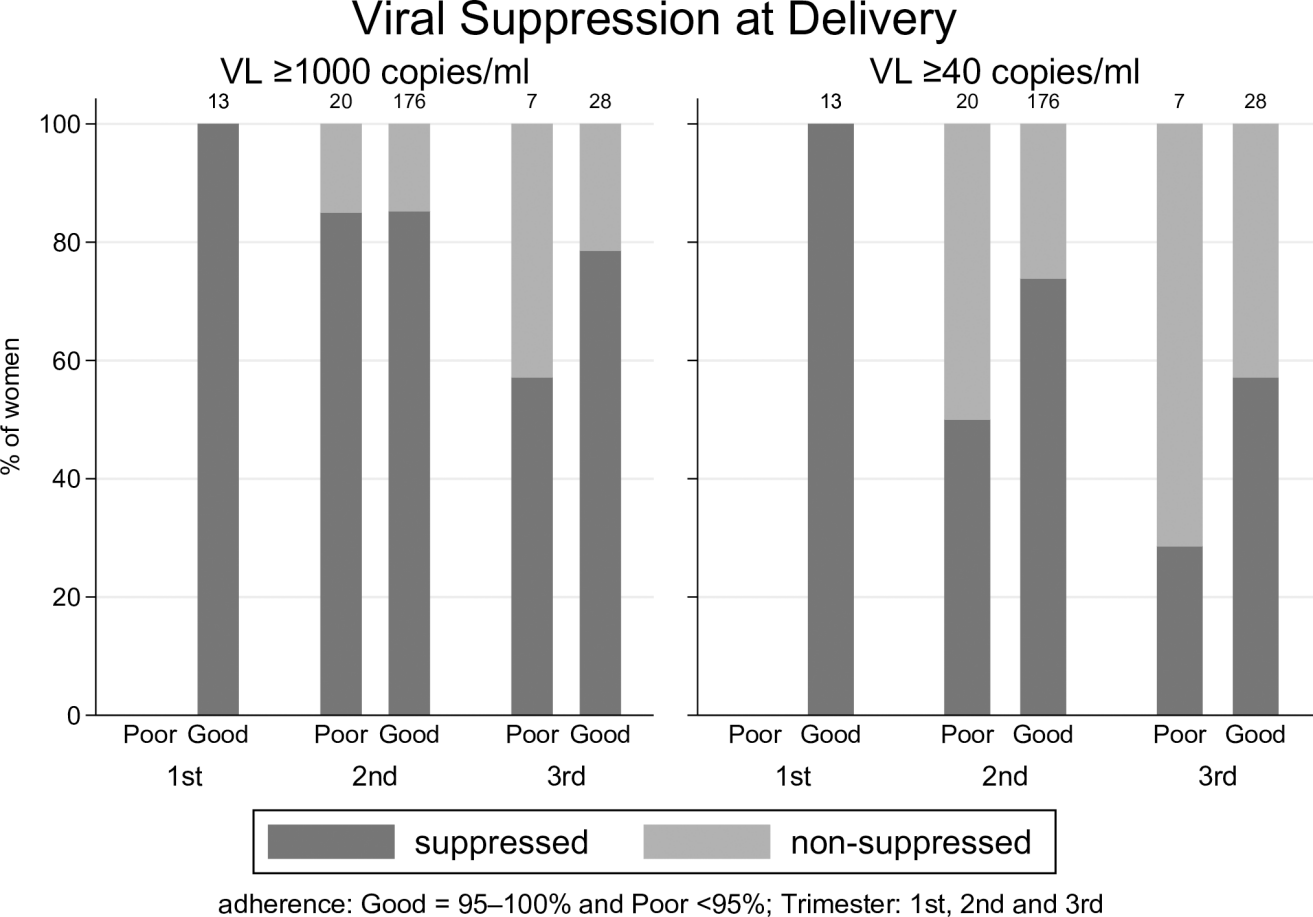
The distribution of women who achieved viral suppression at delivery stratified by level of ART adherence and the trimester at ART initiation.

Information on ART adherence during pregnancy was available for 244 women; 27 women (11.1%) had poor adherence. The proportion of women with poor adherence was higher among women who were on ART for ≤12 weeks (9/50 = 18.0%) than among those who were on ART for 13–35 weeks (18/194 = 9.3%). A higher proportion of women with poor adherence had VL ≥1000 (22.2% vs 14.8%) copies/ml and VL ≥40 copies/ml (55.5% vs 26.7%) at delivery than those with good adherence. The distribution of women who achieved viral suppression at delivery stratified by level of ART adherence and the trimester at ART initiation is shown in Fig 1.

In the primary analysis, women who were on ART for 13–20 weeks (adjusted risk ratio (aRR) = 0.52; 95% CI: 0.36–0.74) or 21–35 weeks (aRR = 0.26; 95% CI: 0.14–0.48) were less likely to have VL ≥40 copies/ml than women who were on ART for ≤12 weeks (Table 4). The risk of VL ≥1000 copies/ml was similarly lower in women who were on ART for 21–35 weeks compared to those who were on ART for ≤12 weeks. The point estimate comparing women who were on ART for 13–20 weeks to those who were on ART for ≤12 weeks suggested a reduced risk of having VL ≥1000 copies/ml at delivery.

**Table 4:** Associations between duration of ART at delivery and viral suppression at delivery.

		Viral suppression at delivery			
Duration of ART		≥1000 copies/ml (N = 40)	<1000 copies/ml (N = 212)	Unadjusted RR (95% CI)	Adjusted RR^†^ (95% CI)
	≤ 12 weeks	15 (26.8)	41 (73.2)	1.00	1.00
	13–20 weeks	19 (15.2)	106 (84.8)	0.57 (0.31, 1.03)	0.67 (0.37, 1.22)
	21–35 weeks	6 (8.5)	65 (91.5)	0.32 (0.13, 0.76)	0.36 (0.15, 0.90)
		≥40 copies/ml (N = 78)	<40 copies/ml (N = 175)		
	≤ 12 weeks	32 (57.1)	24 (42.9)	1.00	1.00
	13–20 weeks	35 (28.0)	90 (72.0)	0.49 (0.34, 0.70)	0.52 (0.36, 0.74)
	21–35 weeks	11 (15.5)	60 (84.5)	0.28 (0.15, 0.50)	0.26 (0.14, 0.48)

^†^Adjusted for age, BMI, baseline CD4 cell count, baseline viral load, education, marital status, parity, and HIV WHO clinical stage

In stratified analysis by category of ART duration, we found that among women on ART for 13–35 weeks, poor adherence was strongly associated with VL ≥40 copies/ml (aRR = 2.39; 95% CI: 1.38–4.15) but not with VL ≥1000 copies/ml (aRR = 1.36; 95% CI: 0.43–4.32) at delivery. However, among women on ART for ≤12 weeks poor adherence was not associated with VL ≥1000 copies/ml (aRR = 2.96; 95% CI: 0.50–17.54) or VL ≥40 copies/ml (aRR = 1.09; 95% CI: 0.48–2.47) at delivery.

Women who were on ART for 13–35 weeks with poor adherence had a higher risk of VL ≥40 copies/ml at delivery (aRR = 2.43; 95% CI: 1.43–4.12) compared to women who were on ART for a similar duration but had good adherence (Table 5). Compared to women who were on ART for 13–35 weeks with good adherence, women who were on ART for ≤12 weeks with good adherence and ≤12 weeks with poor adherence had higher risk of VL ≥40 copies/ml at delivery (aRR = 2.57; 95% CI: 1.72–3.84 and aRR = 2.90; 95% CI: 1.41–5.98, respectively). A similar pattern in risk was observed for VL ≥1000 copies/ml at delivery, although the risk increase compared to women on ART for 13–35 weeks with good adherence was only significant for those on ART for ≤12 weeks with good adherence (aRR = 1.89; 95% CI 1.01–3.54), with a trend towards increased risk seen for those on ART for 13–35 weeks with poor adherence (aRR = 1.30; 95% CI: 0.40–4.21) and those on ART ≤12 weeks with poor adherence (aRR = 2.46; 95% CI: 0.59–10.25).

**Table 5:** Associations between duration of ART at delivery and viral suppression at delivery stratified by levels of ART adherence.

			Viral Suppression at delivery			
Duration of ART		ART adherence*	≥1000 copies/ml (N = 38)	<1000 copies/ml (N = 206)	Unadjusted RR (95% CI)	Adjusted RR^†^ (95% CI)
13–35 weeks		Good	21 (11.9)	155 (88.1)	1.0	1.0
		Poor	3 (16.7)	15 (83.3)	1.40 (0.46, 4.24)	1.30 (0.40, 4.21)
≤ 12 weeks		Good	11 (26.8)	30 (73.2)	2.45 (1.18, 4.29)	1.89 (1.01, 3.52)
		Poor	3 (33.3)	6 (66.7)	2.79 (1.02, 7.68)	2.46 (0.59,10.25)
			≥40 copies/ml (N = 73)	<40 copies/ml (N = 171)		
13–35 weeks		Good	36 (20.5)	140 (79.5)	1.0	1.0
		Poor	9 (50.0)	9 (50.0)	2.44 (1.41, 4.23)	2.43 (1.43, 4.12)
≤ 12 weeks		Good	22 (53.7)	19 (46.3)	2.62 (1.74, 3.95)	2.57 (1.72, 3.84)
		Poor	6 (66.7)	3 (33.3)	3.26 (1.89, 5.63)	2.90 (1.41, 5.98)

^†^Adjusted for age, BMI, baseline CD4 cell count, baseline viral load, education, marital status, parity, and HIV WHO clinical stage

* Good: 95–100%, Poor: <95%

When ART duration was considered as a continuous exposure, there was evidence of a reduced risk of having non-suppressed viral load at delivery with longer duration of ART during pregnancy. For each additional week on ART, the adjusted risk of having VL >1000 copies/ml and VL ≥40 copies/ml at delivery decreased by 0.049 (95% CI: -0.098 –-0.001) and 0.086 (95% CI: -0.121 –-0.052) on the log scale, respectively.

## Discussion

The results from our prospective cohort study of ART-naïve pregnant women who started ART during pregnancy show that being on ART for more than 12 weeks before delivery (starting ART before the third trimester) is associated with suppressed VL at the time of delivery. The association between duration of ART and viral suppression varied with level of treatment adherence. Specifically, being on ART for 13–35 weeks coupled with good adherence to treatment was associated with the greatest reduction in the risk of non-suppressed VL at delivery. These findings are important because they highlight the need for not only initiating ART early in pregnancy, but also being adherent to therapy if ART for PMTCT is to be effective in achieving viral suppression at delivery.

The proportion of pregnant women (15.9%) in this study who did not achieve VL <1000 copies/ml around the time of delivery is concerning. Viral suppression during pregnancy is one of the goals for preventing MTCT. The current WHO virological criterion for treatment failure is VL ≥1,000 copies/ ml.[16] High viral load, particularly if >1000 copies/ml, is an important predictor of fetal HIV transmission, [1–3, 17] and most fetal infections occur during the second or third trimester.[18–20] If poor adherence is not detected and remedied immediately, treatment failure during pregnancy can lead to the development of HIVDR strains that can subsequently be transmitted to the infant.[13]

However, a majority of women (73%) who were on ART for ≤12 weeks in this study had VL <1000 copies/ml at delivery despite the short period that they were on therapy. This finding is consistent with the results from a cohort of pregnant women who were initiated or restarted on ART in United States of America (USA).[21] Most of the women in the USA cohort achieved viral suppression within 25 days after ART initiation. Considering that a substantial number of women report for their first ANC visit late in pregnancy, the large proportion of pregnant women who achieve viral suppression despite starting ART late in pregnancy is encouraging. This information is also important for policymakers as they consider the optimal duration for ART during pregnancy and balance the need for viral suppression with concern for adverse birth outcomes among women on long durations of ART.

Poor adherence rate among women who were on ART for ≤12 weeks was approximately double of those who were on ART for 13–35 weeks (18.0% vs 9.3%). Women who are on ART for ≤12 weeks are late ANC initiators who have limited time on ART before delivery. Late ANC initiators who are also poor adherers to ART represent women that have poor access to health care or low trust in the health care system. However, with good adherence, these late initiators can achieve VL <1000 copies/ml by the time of delivery.[21] Focused support should be given to these women to not only bring them into care early in their pregnancy but also keep them in care if viral suppression at delivery is to be achieved.

Our finding that longer duration of ART during pregnancy is associated with reduced risk of non-suppressed VL corroborates results reported by other studies looking at viral suppression at delivery. Among pregnant women who enrolled in an observational cohort study in Europe, the percent of women reaching undetectable VL at delivery increased with increase in the duration of ART at the time of delivery.[22] In Benin, the probability of having undetectable VL at delivery increased 4-fold if the ART treatment during pregnancy lasted at least 8 weeks.[23] In South Africa, pregnant women starting ART in the third trimester were at greater risk of failing to achieve viral suppression at delivery.[24]

Studies looking at the association between duration of ART during pregnancy and perinatal HIV transmission have also found that starting ART early in pregnancy is necessary for PMTCT. A retrospective cohort analysis of pregnant HIV-infected women attending public antenatal care clinics in Zambia showed decreased odds of HIV transmission if the mother was on therapy for at least 13 weeks.[25] Studies modeling the effect of duration of combination ART (cART) in HIV-infected women who started cART during pregnancy reported a decline in the probability of MTCT[26] and reduced odds of MTCT[27] with each additional week of treatment.

In this study, we have shown that the risk of non-suppressed VL at delivery among ART-naïve pregnant women who started ART during pregnancy decreases with increase in the duration of ART in the era of Option B+. Among women who were on ART for a similar duration, the risk of having non-suppressed VL was lower in women with good adherence to treatment compared to those with poor adherence. These findings suggest that viral suppression at delivery can be achieved if pregnant women not only attend ANC early in pregnancy but also adhere to ART throughout the pregnancy period. Early attendance at ANC is critical for new ART initiators if maximum time on therapy is to be realized. Good adherence to ART ensures adequate therapeutic levels of ART necessary to suppress viral replication.[28]

Development of ART-related side effects such as nausea, vomiting, diarrhea, and loss of appetite [29–32] contribute to poor adherence among pregnant women, depending on the ART regimen. In this study, we did not explore the reasons behind poor adherence to treatment. We also do not rule out the presence of other non-ART related factors that influence poor adherence. Regardless of the reasons for poor adherence to ART, poor adherence during pregnancy increases the burden of HIV through the development of drug resistant strains,[33] increased MTCT, and maternal morbidity and mortality.[34, 35]

Our definition of ART non-adherence was based on reported number of pills missed over the pregnancy period. Self-reported adherence can result in adherence misclassification due to recall bias or tendency of individuals to overstate adherence.[36] However, we do not expect the use of self-reported adherence to have influenced our findings. In this study, we collected redundant information to verify the accuracy of self-reported adherence. We collected the self-reported number of pills missed yesterday, 2 days ago, 2 weeks ago, and 30 days ago. In addition, we recorded the number of pills brought to the clinic, self-reported number of pills left at home, and number of pills dispensed during every clinic visit.

The VL at delivery among women who had poor adherence to ART may depend on the period when they had poor adherence to treatment. In this analysis, we did not take into consideration the timing of poor adherence for women who started ART early in pregnancy. When ART adherence is good early in pregnancy and poor late in pregnancy, VL levels around the time of delivery can be high due to waning drug levels in the body or the development of ART resistance. The VL levels around the time of delivery can also be high when ART adherence is poor early in pregnancy but good late in pregnancy if ART resistance has developed or the good adherence is not long enough to achieve viral suppression.

Some of our comparisons in stratified analyses and analyses that included ART adherence as an EMM suffered from low precision due to small sample size. The inadequate sample size may have contributed to our strange observation of lack of a significant association between poor adherence and viral suppression within the duration of ART categories. Our study was not powered to assess any EMM. Although ART adherence is a risk factor for viral suppression at delivery, we could not include ART adherence as a confounder of the relationship between duration of ART and viral suppression because ART adherence is not a risk factor for duration of ART but may be a consequence of duration of ART. Nevertheless, our findings around ART adherence highlight the existence of positive synergy between long duration of ART and good adherence in achieving viral suppression at delivery.

Our findings suggest that starting ART early in pregnancy coupled with good adherence to treatment can reduce the risk of non-suppressed VL at delivery. Countries implementing Option B+ should vigorously promote early ANC attendance in pregnancy to facilitate prompt ART initiation for HIV-infected women. Enhanced ART adherence counselling among HIV-infected pregnant women should also be prioritized in the global effort to eliminate HIV vertical transmission.

## References

[1] Mofenson LM, Lambert JS, Stiehm ER, Bethel J, Meyer WA 3rd, Whitehouse J, et al Risk factors for perinatal transmission of human immunodeficiency virus type 1 in women treated with zidovudine. Pediatric AIDS Clinical Trials Group Study 185 Team. The New England journal of medicine. 1999;341(6):385–93. Epub 1999/08/05. doi: 10.1056/NEJM199908053410601 . 10432323 10.1056/NEJM199908053410601

[2] Garcia PM, Kalish LA, Pitt J, Minkoff H, Quinn TC, Burchett SK, et al Maternal levels of plasma human immunodeficiency virus type 1 RNA and the risk of perinatal transmission. Women and Infants Transmission Study Group. The New England journal of medicine. 1999;341(6):394–402. Epub 1999/08/05. doi: 10.1056/NEJM199908053410602 . 10432324 10.1056/NEJM199908053410602

[3] Fawzi W, Msamanga G, Renjifo B, Spiegelman D, Urassa E, Hashemi L, et al Predictors of intrauterine and intrapartum transmission of HIV-1 among Tanzanian women. AIDS (London, England). 2001;15(9):1157–65. Epub 2001/06/21. . 11416718 10.1097/00002030-200106150-00011

[4] Dabis F, Ekpini ER. HIV-1/AIDS and maternal and child health in Africa. Lancet. 2002;359(9323):2097–104. Epub 2002/06/28. doi: 10.1016/S0140-6736(02)08909-2 . 12086778 10.1016/S0140-6736(02)08909-2

[5] Phillips AN, Staszewski S, Weber R, Kirk O, Francioli P, Miller V, et al HIV viral load response to antiretroviral therapy according to the baseline CD4 cell count and viral load. JAMA: the journal of the American Medical Association. 2001;286(20):2560–7. Epub 2001/11/28. . 11722270 10.1001/jama.286.20.2560

[6] Graham SM, Holte SE, Peshu NM, Richardson BA, Panteleeff DD, Jaoko WG, et al Initiation of antiretroviral therapy leads to a rapid decline in cervical and vaginal HIV-1 shedding. AIDS (London, England). 2007;21(4):501–7. Epub 2007/02/16. doi: 10.1097/QAD.0b013e32801424bd . 17301569 10.1097/QAD.0b013e32801424bd

[7] Connor EM, Sperling RS, Gelber R, Kiselev P, Scott G, O'Sullivan MJ, et al Reduction of maternal-infant transmission of human immunodeficiency virus type 1 with zidovudine treatment. Pediatric AIDS Clinical Trials Group Protocol 076 Study Group. The New England journal of medicine. 1994;331(18):1173–80. Epub 1994/11/03. doi: 10.1056/NEJM199411033311801 . 7935654 10.1056/NEJM199411033311801

[8] CDC. Achievements in public health. Reduction in perinatal transmission of HIV infection—United States, 1985–2005. MMWR Morbidity and mortality weekly report. 2006;55(21):592–7. Epub 2006/06/03. . 16741495

[9] Attia S, Egger M, Muller M, Zwahlen M, Low N. Sexual transmission of HIV according to viral load and antiretroviral therapy: systematic review and meta-analysis. AIDS (London, England). 2009;23(11):1397–404. Epub 2009/04/22. doi: 10.1097/QAD.0b013e32832b7dca . 19381076 10.1097/QAD.0b013e32832b7dca

[10] Anglemyer A, Rutherford GW, Horvath T, Baggaley RC, Egger M, Siegfried N. Antiretroviral therapy for prevention of HIV transmission in HIV-discordant couples. The Cochrane database of systematic reviews. 2013;4:CD009153 Epub 2013/05/02. doi: 10.1002/14651858.CD009153.pub3 . 23633367 10.1002/14651858.CD009153.pub3 PMC4026368

[11] Cohen MS, Chen YQ, McCauley M, Gamble T, Hosseinipour MC, Kumarasamy N, et al Prevention of HIV-1 infection with early antiretroviral therapy. The New England journal of medicine. 2011;365(6):493–505. Epub 2011/07/20. doi: 10.1056/NEJMoa1105243 ; PubMed Central PMCID: PMCPMC3200068. 21767103 10.1056/NEJMoa1105243 PMC3200068

[12] Lyons FE, Coughlan S, Byrne CM, Hopkins SM, Hall WW, Mulcahy FM. Emergence of antiretroviral resistance in HIV-positive women receiving combination antiretroviral therapy in pregnancy. AIDS (London, England). 2005;19(1):63–7. Epub 2005/01/01. . 15627034 10.1097/00002030-200501030-00007

[13] Zeh C, Weidle PJ, Nafisa L, Lwamba HM, Okonji J, Anyango E, et al HIV-1 drug resistance emergence among breastfeeding infants born to HIV-infected mothers during a single-arm trial of triple-antiretroviral prophylaxis for prevention of mother-to-child transmission: a secondary analysis. PLoS medicine. 2011;8(3):e1000430 doi: 10.1371/journal.pmed.1000430 21468304 10.1371/journal.pmed.1000430 PMC3066134

[14] CDC. Impact of an innovative approach to prevent mother-to-child transmission of HIV—Malawi, July 2011-September 2012. MMWR Morbidity and mortality weekly report. 2013;62(8):148–51. Epub 2013/03/01. . 23446514 PMC4604864

[15] Schouten EJ, Jahn A, Midiani D, Makombe SD, Mnthambala A, Chirwa Z, et al Prevention of mother-to-child transmission of HIV and the health-related Millennium Development Goals: time for a public health approach. Lancet. 2011;378(9787):282–4. Epub 2011/07/19. doi: 10.1016/S0140-6736(10)62303-3 . 21763940 10.1016/S0140-6736(10)62303-3

[16] WHO. Consolidated Guidelines on the Use of Antiretroviral Drugs for Treating and Preventing HIV Infection Recommendation for a Public Health Approach. Geneva, Switzerland: WHO Press, 2013. 24716260

[17] Jourdain G, Mary JY, Coeur SL, Ngo-Giang-Huong N, Yuthavisuthi P, Limtrakul A, et al Risk factors for in utero or intrapartum mother-to-child transmission of human immunodeficiency virus type 1 in Thailand. The Journal of infectious diseases. 2007;196(11):1629–36. Epub 2007/11/17. doi: 10.1086/522009 . 18008246 10.1086/522009

[18] Soeiro R, Rubinstein A, Rashbaum WK, Lyman WD. Maternofetal transmission of AIDS: frequency of human immunodeficiency virus type 1 nucleic acid sequences in human fetal DNA. The Journal of infectious diseases. 1992;166(4):699–703. Epub 1992/10/01. . 1527405 10.1093/infdis/166.4.699

[19] Ehrnst A, Lindgren S, Dictor M, Johansson B, Sonnerborg A, Czajkowski J, et al HIV in pregnant women and their offspring: evidence for late transmission. Lancet. 1991;338(8761):203–7. Epub 1991/07/27. . 1676777 10.1016/0140-6736(91)90347-r

[20] Courgnaud V, Laure F, Brossard A, Bignozzi C, Goudeau A, Barin F, et al Frequent and early in utero HIV-1 infection. AIDS research and human retroviruses. 1991;7(3):337–41. Epub 1991/03/01. doi: 10.1089/aid.1991.7.337 . 2064830 10.1089/aid.1991.7.337

[21] Aziz N, Sokoloff A, Kornak J, Leva NV, Mendiola ML, Levison J, et al Time to viral load suppression in antiretroviral-naive and -experienced HIV-infected pregnant women on highly active antiretroviral therapy: implications for pregnant women presenting late in gestation. BJOG: an international journal of obstetrics and gynaecology. 2013;120(12):1534–47. Epub 2013/08/09. doi: 10.1111/1471-0528.12226 . 23924192 10.1111/1471-0528.12226

[22] Patel D, Cortina-Borja M, Thorne C, Newell ML. Time to undetectable viral load after highly active antiretroviral therapy initiation among HIV-infected pregnant women. Clinical infectious diseases: an official publication of the Infectious Diseases Society of America. 2007;44(12):1647–56. Epub 2007/05/23. doi: 10.1086/518284 . 17516411 10.1086/518284

[23] Denoeud-Ndam L, Fourcade C, Ogouyemi-Hounto A, Azon-Kouanou A, d'Almeida M, Azondekon A, et al Predictive factors of plasma HIV suppression during pregnancy: a prospective cohort study in Benin. PloS one. 2013;8(3):e59446 Epub 2013/04/05. doi: 10.1371/journal.pone.0059446 ; PubMed Central PMCID: PMCPMC3598754. 23555035 10.1371/journal.pone.0059446 PMC3598754

[24] Myer L, Phillips TK, McIntyre JA, Hsiao NY, Petro G, Zerbe A, et al HIV viraemia and mother-to-child transmission risk after antiretroviral therapy initiation in pregnancy in Cape Town, South Africa. HIV medicine. 2017;18(2):80–8. Epub 2016/06/30. doi: 10.1111/hiv.12397 . 27353189 10.1111/hiv.12397

[25] Chibwesha CJ, Giganti MJ, Putta N, Chintu N, Mulindwa J, Dorton BJ, et al Optimal time on HAART for prevention of mother-to-child transmission of HIV. Journal of acquired immune deficiency syndromes (1999). 2011;58(2):224–8. Epub 2011/06/29. doi: 10.1097/QAI.0b013e318229147e ; PubMed Central PMCID: PMCPMC3605973. 21709566 10.1097/QAI.0b013e318229147e PMC3605973

[26] Townsend CL, Byrne L, Cortina-Borja M, Thorne C, de Ruiter A, Lyall H, et al Earlier initiation of ART and further decline in mother-to-child HIV transmission rates, 2000–2011. AIDS (London, England). 2014;28(7):1049–57. Epub 2014/02/26. doi: 10.1097/qad.0000000000000212 . 24566097 10.1097/QAD.0000000000000212

[27] Hoffman RM, Black V, Technau K, van der Merwe KJ, Currier J, Coovadia A, et al Effects of highly active antiretroviral therapy duration and regimen on risk for mother-to-child transmission of HIV in Johannesburg, South Africa. Journal of acquired immune deficiency syndromes (1999). 2010;54(1):35–41. Epub 2010/03/11. doi: 10.1097/QAI.0b013e3181cf9979 ; PubMed Central PMCID: PMCPMC2880466. 20216425 10.1097/QAI.0b013e3181cf9979 PMC2880466

[28] Paterson DL, Swindells S, Mohr J, Brester M, Vergis EN, Squier C, et al Adherence to protease inhibitor therapy and outcomes in patients with HIV infection. Annals of internal medicine. 2000;133(1):21–30. Epub 2000/07/06. . 10877736 10.7326/0003-4819-133-1-200007040-00004

[29] Chesney M. Adherence to HAART regimens. AIDS patient care and STDs. 2003;17(4):169–77. Epub 2003/05/10. doi: 10.1089/108729103321619773 . 12737640 10.1089/108729103321619773

[30] Phillips T, Cois A, Remien RH, Mellins CA, McIntyre JA, Petro G, et al Self-Reported Side Effects and Adherence to Antiretroviral Therapy in HIV-Infected Pregnant Women under Option B+: A Prospective Study. PloS one. 2016;11(10):e0163079 Epub 2016/10/21. doi: 10.1371/journal.pone.0163079 ; PubMed Central PMCID: PMCPMC5070813. 27760126 10.1371/journal.pone.0163079 PMC5070813

[31] Duran S, Spire B, Raffi F, Walter V, Bouhour D, Journot V, et al Self-reported symptoms after initiation of a protease inhibitor in HIV-infected patients and their impact on adherence to HAART. HIV clinical trials. 2001;2(1):38–45. Epub 2001/10/09. doi: 10.1310/R8M7-EQ0M-CNPW-39FC . 11590513 10.1310/R8M7-EQ0M-CNPW-39FC

[32] Ebuy H, Yebyo H, Alemayehu M. Level of adherence and predictors of adherence to the Option B+ PMTCT programme in Tigray, northern Ethiopia. International journal of infectious diseases: IJID: official publication of the International Society for Infectious Diseases. 2015;33:123–9. Epub 2014/12/23. doi: 10.1016/j.ijid.2014.12.026 . 25529555 10.1016/j.ijid.2014.12.026

[33] Bangsberg DR, Acosta EP, Gupta R, Guzman D, Riley ED, Harrigan PR, et al Adherence-resistance relationships for protease and non-nucleoside reverse transcriptase inhibitors explained by virological fitness. AIDS (London, England). 2006;20(2):223–31. Epub 2006/03/03. doi: 10.1097/01.aids.0000199825.34241.49 . 16511415 10.1097/01.aids.0000199825.34241.49

[34] Bangsberg DR, Perry S, Charlebois ED, Clark RA, Roberston M, Zolopa AR, et al Non-adherence to highly active antiretroviral therapy predicts progression to AIDS. AIDS (London, England). 2001;15(9):1181–3. Epub 2001/06/21. . 11416722 10.1097/00002030-200106150-00015

[35] Garcia de Olalla P, Knobel H, Carmona A, Guelar A, Lopez-Colomes JL, Cayla JA. Impact of adherence and highly active antiretroviral therapy on survival in HIV-infected patients. Journal of acquired immune deficiency syndromes (1999). 2002;30(1):105–10. Epub 2002/06/06. . 12048370 10.1097/00042560-200205010-00014

[36] Berg KM, Arnsten JH. Practical and conceptual challenges in measuring antiretroviral adherence. Journal of acquired immune deficiency syndromes (1999). 2006;43 Suppl 1:S79–87. Epub 2006/11/30. doi: 10.1097/01.qai.0000248337.97814.66 ; PubMed Central PMCID: PMCPMC2866146. 17133207 10.1097/01.qai.0000248337.97814.66 PMC2866146

